# Radiological Assessment and Therapeutic Evaluation in Hepatocellular Carcinoma: Differentiation and Treatment Response with Japanese Guidelines

**DOI:** 10.3390/cancers17010101

**Published:** 2024-12-31

**Authors:** Masakatsu Tsurusaki, Keitaro Sofue, Takamichi Murakami, Noboru Tanigawa

**Affiliations:** 1 Department of Radiology, Kansai Medical University Medical Center, Moriguchi 570-8503, Osaka, Japan; 2 Department of Radiology, Kobe University Graduate School of Medicine, Kobe 650-0017, Hyogo, Japan; keitarosofue@yahoo.co.jp (K.S.); murataka@med.kobe-u.ac.jp (T.M.); 3 Department of Radiology, Kansai Medical University, Hirakata 573-1010, Osaka, Japan; tanigano@hirakata.kmu.ac.jp

**Keywords:** hepatocellular carcinoma, dynamic CT, RECIST, mRECIST, RECICL, treatment response criteria, Wnt/β-catenin mutation/activation, Gd-EOB-DTPA enhanced MRI

## Abstract

The liver is supplied by a dual blood flow system consisting of the portal vein and hepatic artery. Imaging techniques for diagnosing hepatocellular carcinoma (HCC) have been developed along with blood flow imaging, which visualizes the amount of arterial and portal blood flow. The diagnosis of HCC differentiation is important for early-stage liver cancer screening and determination of treatment strategies. Dynamic computed tomography/magnetic resonance imaging (MRI) includes blood flow imaging and MRI with contrast-enhanced ultrasound and liver-specific contrast agents are used in combination. In addition, unlike the Response Evaluation Criteria in Solid Tumors (RECIST) (version 1.1), which is the standard for determining treatment efficacy for solid tumors in general, tumor necrosis is generally considered a treatment effect in HCC, and the modified RECIST and Liver Cancer Direct Effectiveness Criteria (RECICL) are widely used. Familiarity with the definitions, criteria, and potential challenges of the mRECIST and RECICL is essential for their effective application in clinical practice. This review integrates the latest advancements in systemic treatments and imaging techniques, including the role of LI-RADS and updates on molecular-targeted therapies such as regorafenib, supported by some systematic review and meta-analysis.

## Simple Summary

Unlike RECIST (version 1.1), which is the standard for assessing the treatment efficacy for solid tumors, tumor necrosis is considered a marker of the treatment effect in HCC. mRECIST, which considers tumor necrosis, and the Liver Cancer Treatment Direct Effectiveness Criteria (RECICL) are widely used. There are notable differences between one-dimensional and two-dimensional measurements in mRECIST and RECICL, and their practicality and usefulness are controversial. After fully understanding the characteristics and problems of each method, diagnostic imaging physicians must focus on the imaging modality and criteria that should be used to determine the effectiveness of the method. Furthermore, the inclusion of LI-RADS provides a standardized framework for evaluating HCC and supports consistent diagnostic and therapeutic decision-making. Given the availability of several drugs, predicting the efficacy of systemic chemotherapy for HCC is currently a clinical topic. However, the role of CT is limited. Recently, attempts have been made to detect Wnt/β-catenin mutation/activation in HCC using Gd-EOB-DTPA enhanced MRI (EOB-MRI), and future developments are expected in terms of predicting efficacy. For instance, regorafenib has demonstrated survival benefits in patients previously treated with sorafenib, as highlighted in a recent systematic review and meta-analysis. Additionally, atezolizumab combined with bevacizumab has emerged as a first-line treatment for unresectable hepatocellular carcinoma.

## 1. Introduction

Since the advent of computed tomography (CT) and magnetic resonance imaging (MRI), imaging-based diagnosis of HCC has relied on three main modalities: CT, MRI, and conventional ultrasound. Based on the general principle that the liver is supplied by dual blood flow, with portal and arterial blood flows predominating in the liver parenchyma, whereas typical HCCs derive their blood supply exclusively from the arteries, imaging techniques that visualize arterial and portal blood flow have been integral to HCC diagnosis since the early days of CT and MRI. Dynamic imaging studies, including the arterial- and portal-dominant phases, have been performed using imaging over time after the injection of contrast media. The ultimate forms of CT arterial portography and CT hepatic arteriography have been developed, and several excellent studies have been conducted in Japan. These hemodynamic imaging techniques form the basis for HCC diagnosis.

Multi-detector row CT (MDCT) is currently in clinical use, dramatically reducing scanning time. Perfusion CT was once in the limelight as a diagnostic method for liver blood flow; however, it is not widely used for general examinations because of its complicated nature and the recent increase in X-ray exposure. With the exception of special imaging methods, such as perfusion CT, it is easy to separate the arterial and portal phases with MDCT with 64 or more rows, and post-contrast three-phase imaging is commonly used by adding an equilibrium phase to the arterial phase. In fact, because of the fast scan time, it is important to optimize the imaging timing after contrast injection and the injection method, such as the amount, concentration, and injection speed of the contrast agent, and implement an appropriate imaging protocol for the diagnosis of HCC. Recent technical topics in the CT diagnosis of HCC are discussed in other sections. This section highlights the fundamental principles and recent advancements in HCC differential diagnosis and the role of imaging in the pre- and post-treatment assessment of advanced HCC.

## 2. Diagnostic Efficacy of Dynamic CT in HCC

**Figure 1:**
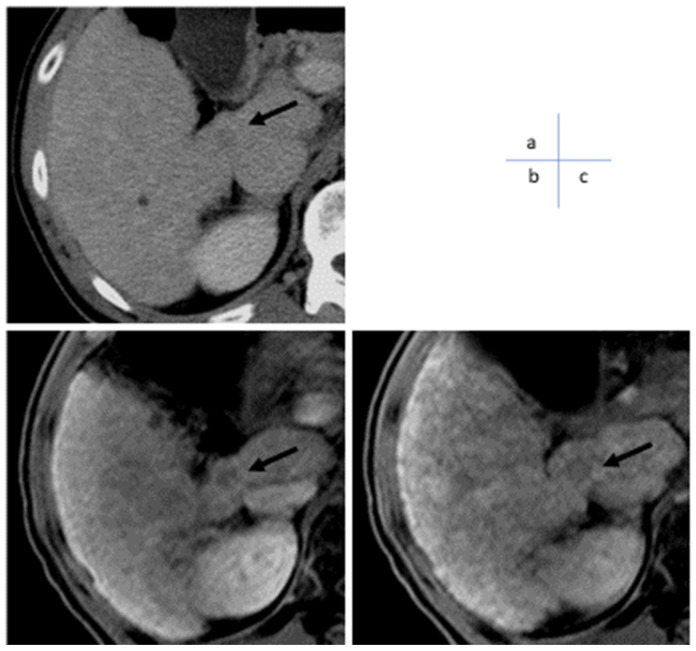
A 60s (oligometastatic) small liver cancer. (**a**) Contrast-enhanced CT (portal vein phase) showed a hypovascular nodule of about 10 mm at the S1/7 border (arrow). (**b**) EOB contrast-enhanced MRI (arterial phase) showed no arterial staining within the nodule, suggesting a hypovascular HCC (arrow). (**c**) EOB contrast-enhanced MRI (hepatocellular phase) showed decreased EOB uptake in the nodule (arrow), which was generally considered borderline lesion to early hepatocellular carcinoma; therefore, the patient was followed-up for polycythemia vera.

**Figure 2:**
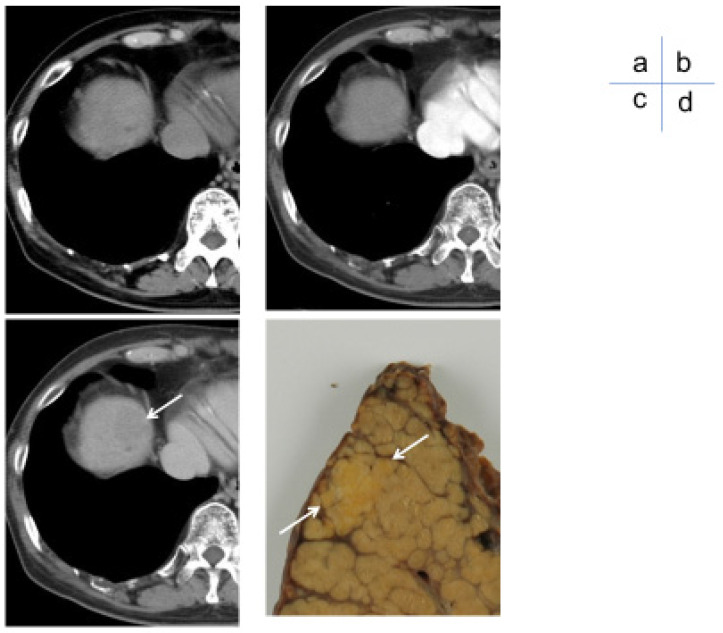
A 50s (oligometastatic) small liver cancer. (**a**) Simple CT showing a slightly hypodense mass in S8 with an indistinct border. (**b**) Dynamic CT arterial phase shows no contrast effect inside the mass. (**c**) In the dynamic CT equilibrium phase, the mass was slightly less dense than the surrounding liver parenchyma and could barely be identified (arrow). (**d**) Resected specimen showed hepatocellular carcinoma with indistinct nodular borders (highly differentiated) (arrow).

The diagnostic utility of dynamic CT/MRI for detecting HCC is well established. The latest 2021 guidelines for HCC treatment [1] state that “Dynamic CT, dynamic MRI, or contrast-enhanced ultrasound is recommended for the diagnosis of typical HCC with EOB-MRI being recommended if either is feasible”. The statement “EOB-MRI is strongly recommended, grade A” leaves no doubt as to its usefulness, at least for the diagnosis of classic HCC. The guidelines recommend follow-up for high-risk patients, mainly for atypical tumors less than 1 cm in arterial phase that are not darkened by arterial phase and for other additional tests or biopsy if the tumor is 1.5 cm or larger but presents atypical imaging findings. These evidence-based guidelines make sense (Supplemental Figure S1). A meta-analysis comparing the diagnostic performances of Gd-EOB-DTPA-enhanced MRI (EOB-MRI), dynamic CT, and dynamic MRI revealed that the estimated sensitivities of EOB-MRI and CE-CT were 0.881 (95% confidence interval [CI] = 0.766, 0.944) and 0.713 (95% CI = 0.577, 0.577), respectively, (0.577, 0.819) and estimated specificity was 0.926 (95% CI = 0.829, 0.97) and 0.918 (95% CI = 0.829, 0.963), respectively. However, these differences were not statistically significant. However, when restricted to studies involving patients with small lesions, EOB-MRI was superior to CE-CT, with estimated sensitivities of 0.919 (95% CI = 0.834–0.962) and 0.637 (95% CI = 0.565–0.704) and an estimated specificity of 0.936 (95% CI = 0.882–0. 966) and 0.971 (95% CI = 0.937, 0.987), respectively [2]. Another meta-analysis confirmed that EOB-MRI showed a significantly higher sensitivity than CT (0.85 vs. 0.68) and that the specificity did not differ between the two (0.94 vs. 0.93) (area under the receiver operating characteristic curve 0.79 vs. 0.46) [3]. In our multicenter study of surgical cases, the sensitivity of EOB-MRI and dynamic MD-CT were 0.83 and 0.70, respectively; 0.58 and 0.28 for ≤1 cm; and 0.84 and 0.73 for 1–2 cm, all significantly superior to EOB-MRI, while for ≥2 cm, the sensitivity was 0.97 and 0.93 and 97 and 0.93, respectively, which were not statistically significant [4] (Figure 1 and Figure 2). Although the detection sensitivity of CT for small HCC remains controversial, it has a high detection sensitivity for hypervascular classical hepatocellular carcinoma of 2 cm or larger, which is the main target of treatment, and almost 100% of HCCs presenting typical findings with current dynamic CT or dynamic MRI can be diagnosed. This provides evidence that imaging of HCC with a focus on hemodynamics is extremely useful. MDCT is widely used in most facilities and has advantages over MRI, such as stable image quality and shorter examination time. Each scan was only a few seconds long and there was little deterioration in image quality, even in cases where respiratory arrest was not possible. Its diagnostic performance is comparable to that of MRI, except for small lesions. Dynamic computed tomography (CT) plays a significant role in diagnosing HCC.

## 3. Role of Dynamic CT/MRI in Differentiating Tumor Grades

**Figure 3:**
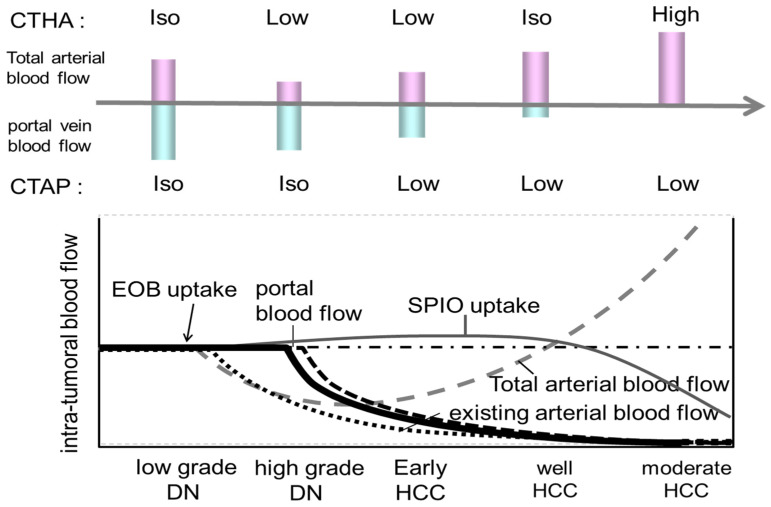
Borderline lesion—HCC imaging (illustration).

**Figure 4:**
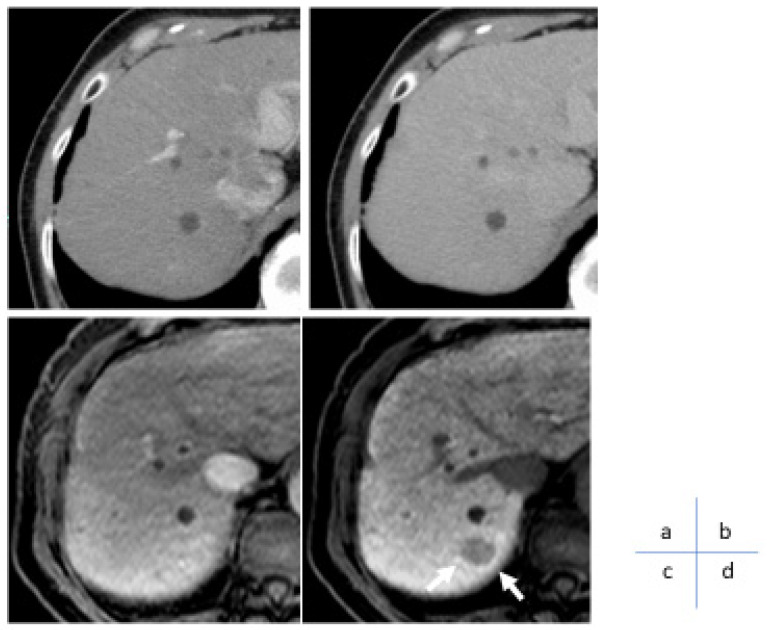
(**a**) Small liver cancer in the 60s dynamic CT arterial phase. (**b**) No mass is observed in equilibrium phase. (**c**) Gd-EOB-DTPA contrast-enhanced arterial-phase MRI image showing no obvious arteriographic enhancement. (**d**) The nodule is clearly noted on the Gd-EOB-DTPA contrast-enhanced MRI hepatocellular phase as a zone of decreased EOB uptake and is suspected to be a highly differentiated hepatocellular carcinoma (arrow), which was subsequently diagnosed as a highly differentiated hepatocellular carcinoma in the surgical specimen.

Since the 1990s, studies have aimed at diagnosing early-stage well-differentiated HCC, known as oligemic HCC. The evolution of contrast-enhanced MRI diagnoses has centered on the use of liver-specific MRI contrast agents such as SPIO (super paramagnetic iron oxide) [5] and Gd-EOB-DTPA [6,7]. When a small nodule is identified via ultrasound, intranodular blood flow is evaluated by dynamic CT/MRI, taking advantage of the difference in arterial and portal blood flow supply between the hepatic nodule and the surrounding liver parenchyma during the multistage development of HCC [8] (Figure 3). However, highly atypical nodules are those that partially show cell density more than twice that of the surrounding liver or have slight structural atypia. There is an overlap in the diagnosis of early-stage HCC and atypical nodules based on intranodular blood flow assessment, limiting differentiation by blood flow between the two. Therefore, at present, the diagnosis of borderline lesions and early HCC is limited to dynamic CT alone, as the diagnosis of oligo- (nonhemolytic) nodules in the hepatocellular phase of Gd-EOB-DTPA contrast-enhanced MRI best reflects differentiation [6] (Figure 4). Recently, however, it has been reported [7] that among non-hypervascularized nodules showing low signal intensity in the hepatocellular phase, 44% are advanced HCC, 20% are early-stage HCC, 27.5% are highly differentiated nodules, and 8% are low-grade dysplastic and regenerative nodules; considerable overlap exists between these that should be noted. There is no clear evidence of the advantages and disadvantages of biopsy and treatment of non-hematopoietic lesions, including borderline lesions and early-stage HCC. According to recent expert opinions regarding the guidelines for the treatment of HCC [1] and other carcinomas, biopsy at the initial presentation is undesirable in view of the balance between its invasiveness and the benefit obtained and should be followed up with the addition of a second contrast scan or imaging studies.

A meta-analysis by Suh et al. found that in patients with chronic liver disease, the rate of polycythemia vera of non-polycythemia detected by EOB-MRI in the hepatocyte phase was 18% at 1 year, 25% at 2 years, and 30% at 3 years [9]. Therefore, the cumulative hypervascularization rate of non-hypervascularized lesions is high and should not be neglected. Regular follow-up for hypervascularization, including dynamic CT/MRI, is important.

## 4. Treatment Response Assessment: Response Evaluation Criteria in Solid Tumors (RECIST), Modified RECIST (mRECIST), and Liver Cancer Direct Effectiveness Criteria (RECICL) Criteria (Table 1)

In 2000, the RECIST guidelines were introduced as an alternative to the conventional WHO criteria for assessing treatment responses in solid tumors [10]. RECIST primarily evaluates the treatment response by measuring changes in tumor length in one dimension. Owing to its simplicity and versatility, many clinical studies have used this criterion to evaluate outcomes, and it has now been further simplified and is widely used in RECIST (version 1.1) [11]. Although HCC and other types of liver cancer are carcinomas that can be treated with various local therapies to induce necrosis, RECIST does not consider such necrosis as “effective” but only tumor shrinkage as a measure of efficacy and is therefore considered inappropriate as a criterion for determining the efficacy of treatment for liver cancer. For example, studies have shown no correlation between tumor shrinkage (as measured by the WHO criteria or RECIST) and pathological necrosis following transarterial chemoembolization (TACE) treatment; however, there is a discrepancy between the necrotic effect and effectiveness criteria for the local treatment characteristics of liver cancer, such as TACE. Furthermore, it is well known that classic HCC is stained in the arterial phase and has low absorption (washout) in the portal vein phase on dynamic contrast-enhanced CT. However, with the advent of molecular-targeted therapeutic drugs that have recently been introduced, there have been cases in which tumor staining disappeared, while the tumor size did not change. In 2010, Lencioni et al. proposed the mRECIST, which evaluates the diameter of a darkened tumor area [12]. The main idea was that in HCC, which is characterized by hypervascularity, the disappearance of tumor staining was considered the disappearance of a viable tumor. In the treatment of HCC, in which blood flow is important for evaluation, it seems appropriate to evaluate tumor staining, which has already been used as an efficacy criterion in many clinical trials and is increasingly used in daily practice.

**Table 1:** Comparison of response evaluation criteria.

	RECIST 1.1	mRECIST	RECICL 2021
Target lesion	Target lesion (five lesions, max two lesions per organ)	Target lesion (10 lesions, maximum five lesions per organ)	Maximum two lesions per organ (but not more than three lesions in the liver) Up to five lesions
Evaluation Method	Unidirectional measurement (Change in total longest diameter)	Unidirectional measurement (Change in total longest diameter. However, the indistinct area in contrast-enhanced CT is measured as necrosis.)	Two-way measurement (Change in the product of the longest diameter and the diameter perpendicular to it. Lipiodol deposition areas without stained areas or washout in CT are measured as necrosis.)
Comprehensive evaluation judgment method	All targets before treatment. The sum of the longest diameter of the lesion is All targets after treatment Sum and ratio of longest diameter of lesion comparison	All target diseases before treatment. of the longest diameter of the non-necrotic part of the deformity. Sums for all post-treatment Target lesion (non-necrotic area) Sum of longest diameter	The sum of the products of the longest diameters of all target lesions and their orthogonal diameters before treatment is the baseline area sum. The sum of the products of the longest diameter and orthogonal diameters of necrotic/reduced lesions of all target lesions after treatment is the sum of area summation, and the difference from the baseline area summation is divided by the baseline area summation and expressed as “Overall TE” to contribute to the calculation of the overall evaluation judgment.
CR	Disappearance of all target lesions (Lymph nodes less than 10 mm in short diameter)	Disappearance of all tumor staining	100% necrosis effect or 100% shrinkage
PR	Decreased by more than 30	Viable lesion diameter reduction of 30% or more	Necrotic effect 50% to less than 100% or reduction of 50% to less than 100%
SD	PR, effects other than PD	PR, effects other than PD	PR, effects other than PD
PD	Increase of more than 20% or appearance of new lesions	Increase in the sum of diameters of viable lesions by more than 20% or appearance of new lesions	50% tumor growth (excluding necrotic areas due to treatment) or appearance of new lesions

**Figure 5:**
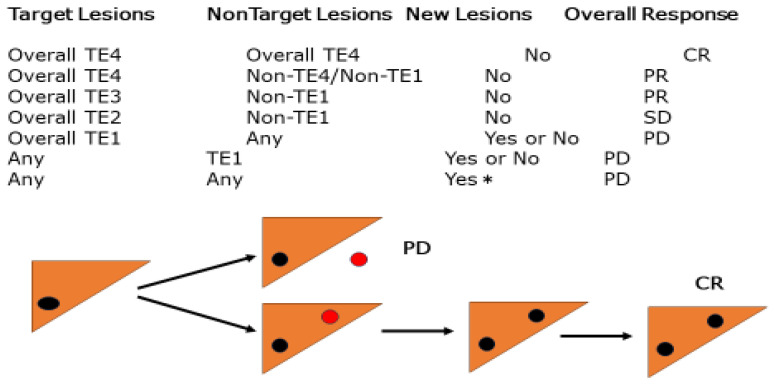
Overall response evaluation by the Response Evaluation Criteria in Cancer of the Liver (RECICL). * TE: treatment effect; CR: complete response; PD: progressive disease; PR: partial response; SD: stable disease; Red dot: new lesion; Black dot: treated lesion.

Other criteria such as the RECICL of the Japanese Association for the Study of Liver Cancer and the Choi Criteria have been proposed to assess the therapeutic efficacy of imatinib for GIST [13,14,15]. The basic concepts of RECICL, as described in a previous report [16], are (1) concise and fully applicable in daily practice, (2) a criterion that can withstand international use, and (3) a criterion for determining therapeutic efficacy, mainly for local ablation therapy (ethanol injection therapy, microwave coagulation therapy, and radiofrequency ablation therapy) and transarterial catheterization. The effectiveness of tumor necrosis/reduction, as evaluated by mRECIST and RECICL, is based on local treatment, not systemic chemotherapy, and is routinely used in Japan. Therefore, it is important to evaluate tumor necrosis/shrinkage using such criteria, as has already been shown in many studies to correlate with prognosis, regardless of local or systemic chemotherapy [17,18,19] (Figure 5).

In the evaluation of the overall response to locoregional therapy, the appearance of a new extrahepatic lesion was assessed as PD, whereas the appearance of only a new intrahepatic lesion was not assessed as PD, and the assessment was made after the next session of treatment. The overall response to the appearance of a new intrahepatic lesion after locoregional therapy may be tentatively described as new CR/PR/SD + intrahepatic lesions.

TE4: 100% tumor necrotizing effect or 100% tumor regression rate; TE4a: necrotic area larger than the original tumor size; TE4b: necrotic area as large as the original tumor size; TE3: tumor necrosis rate < 50% and <100% OR tumor regression rate > 50% and <100%; TE2: effects between TE3 or TE1TE1 ≥ 50% increase in tumor size (excluding the area of treatment-induced necrosis).

**Figure 6:**
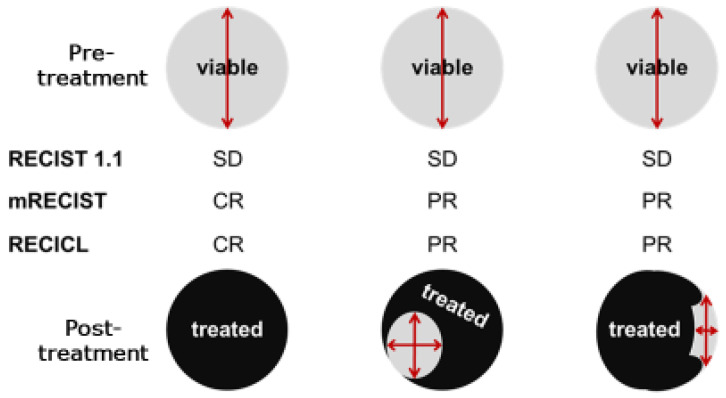
Comparison of response evaluation criteria for liver cancer (illustration).

The RECICL criteria [13] define “necrotic effect” as “an area of low staining seen after treatment on dynamic CT using the rapid intravenous infusion method” and “an area of low staining” means an area of low staining on dynamic CT using the rapid intravenous infusion method, both in the early and equilibrium phases, that is clearly lower than the surrounding liver parenchyma. In other words, the delta of low density caused by rapid intravenous infusion was defined as the area where no increase in CT values was observed before and after contrast. Thus, dynamic CT with rapid intravenous infusion is the optimal imaging method for determining treatment efficacy. The first half of the dynamic phase (arterial-to-intermediate phase) of Gd-EOB-DTPA contrast-enhanced MRI is considered a secondary method. For determining treatment response in systemic chemotherapy with molecular-targeted drugs and recent immunotherapy, the latest guidelines for liver cancer treatment [1] recommend the use of RECIST criteria or modified RECIST criteria for determining treatment response to drug therapy (strong recommendation, strength of evidence A). However, these guidelines do not mention imaging modalities. Furthermore, the aforementioned guideline for the treatment of liver cancer states that “dynamic CT/MRI is recommended for determining the efficacy of local puncture therapy (strong recommendation, strength of evidence B)”, “dynamic CT or dynamic MRI is recommended for determining the efficacy of TACE (strong recommendation, strength of evidence C)”, “dynamic CT or dynamic MRI is recommended for determining the efficacy of TACE (Strong recommendation, strength of evidence A)”, and “Dynamic CT or dynamic MRI is recommended for determining the efficacy of TACE (strong recommendation, strength of evidence B)”. These guidelines describe the imaging modalities used to determine the efficacy of local therapy (Figure 6, Table 2).

The CT/MRI Treatment Response LI RADS^®^ in the American College of Radiology’s LI-RADS v2018 (The Liver Imaging Reporting And Data System) [20] recommends radiofrequency ablation, ethanol injection, cryotherapy, and microwave ablation. The RADS is used to make treatment decisions for local therapies such as radiofrequency ablation, ethanol injection, cryotherapy, microwave ablation, conventional TACE, DEB-TACE, transarterial radioembolization (TARE; not approved in Japan), external-beam radiation therapy, and extracellular fluid contrast media (extracellular fluid) in high-risk patients, including patients with HCC scheduled for liver transplantation. Both pre- and post-extracellular contrast-enhanced CT and MRI or pre- and post-hepatobiliary excretory MRI are recommended for local therapies such as TARE and external radiation therapy in high-risk patients, including liver transplantation candidates.

In CT/MRI Liver Imaging Reporting and Data System (LI-RADS) version 2017 [20] and version 2018 [21], the treatment response algorithm (TRA) takes the tumor viability after locoregional therapy (LRT) into consideration [12,22]. Except for using a feature such as the arterial phase hyperenhancement of lesions in the modified Response Evaluation Criteria in Solid Tumors criteria [12], several new features, including post-treatment “washout”, treatment-specific expected enhancement, and post-treatment enhancement similar to pre-treatment, are observed when evaluating the viability of the treated tumor. Furthermore, this new evaluation system focuses on the variable appearances of individual lesions instead of the patient-level response, providing access to the efficiency of each LRT. Treatment response after LRT is assessed about every 3 months with multiphase contrast-enhanced CT or MRI using extracellular or hepatobiliary contrast agents. The 2017 LI-RADS TRA algorithm divides treatment response into four categories: LI-RADS treatment response (LR-TR) Nonevaluable, Viable, Nonviable, and Equivocal. In the 2017 TRA, the presence or absence of mass-like areas of arterial phase hyperenhancement (APHE) is one of the main criteria for detection of viable disease, although washout and/or enhancement similar to pre-treatment are additional imaging criteria used to assess for tumor viability. However, emerging evidence shows that the expected imaging appearance of treated HCC varies in part depending on the type of LRT, consequently limiting application of the 2017 LI-RADS algorithm for all forms of LRT [23,24,25,26,27,28].

The LI-RADS TRA differs from other TRA systems in that it provides an assessment of individual treatment observations. This is advantageous because in high-risk patients, the progression of the disease is dynamic, with the potential for new, asynchronous lesions to develop anywhere in the liver, and LRT management decisions often need to be made at the lesion level [29]. Since the release of the 2017 algorithm, there have been various non-radiation-based LRT response evaluations, including (hepatic arterial embolization and hepatic arterial chemoembolization) and thermal ablation (microwave ablation and radiofrequency ablation), as well as a wide range of studies in the literature evaluating its performance in assessing the response to various non-radiation LRTs [30,31]. In addition, there are also several studies that have evaluated the accuracy and reading performance of TRA for HCC after local treatment based on radiotherapy (i.e., stereotactic radiotherapy and transarterial embolization) [32,33]. Evaluation of response after local treatment (LRT) for hepatocellular carcinoma (HCC) is extremely important for prognosis prediction.

The LI-RADS TRA provides a standardized approach for image acquisition, interpretation, reporting, and data collection for HCC treated with LRT. It is unique in providing a lesion-level assessment of treated observations while other treatment response systems provide a patient-level assessment (mRECIST, EASL). This is important for this high-risk patient population in which metachronous disease is common, particularly when patients may undergo several different types of LRT over time, based on patient factors, stage of disease, and tumor factors like size and location. Each treated lesion must be evaluated for treatment response to best assess tumor burden for the purposes of bridging and downstaging for liver transplant.

## 5. Dynamic CT/MRI Imaging for Predicting Response to Systemic Chemotherapy in HCC

Sorafenib, a molecular-targeted agent (MTA), was introduced as a first-line treatment for unresectable HCC in Japan in 2009 [34]. Despite numerous developments between 2009 and 2016, no new drugs have been approved.

Clinical trials fail at every turn. However, four drugs (regorafenib, lenvatinib, cabozantinib, and ramucirumab) have been successfully tested in clinical trials since 2017: lenvatinib was approved in 2018 as a first-line treatment to replace sorafenib, followed by second-line treatment after sorafenib failure in June 2017; regorafenib and ramucirumab in June 2019; and cabozantinib in November 2020. These drugs prolong survival by maintaining stable disease or better [35,36,37,38].

Facciorusso et al. [39] reported that the evaluation of the efficacy and safety of lenvatinib compared to sorafenib as first-line systemic therapies for advanced HCC. A systematic review and meta-analysis (SRMA) were conducted, including five studies with 1481 patients. The SRMA study concludes that while lenvatinib improves PFS, OR, and DCR compared to sorafenib, it does not confer a significant survival benefit. The modest impact on OS could be influenced by post-progression treatments and differences in hepatic reserve. These findings suggest that lenvatinib may be a preferable first-line therapy for advanced HCC in terms of disease control, but further research is needed to validate these outcomes and explore long-term survival benefits. Additionally, Facciorusso et al. [40] reported that the efficacy and safety of regorafenib as a second-line treatment for HCC following sorafenib failure. A meta-analysis of one randomized controlled trial and seven non-randomized studies, including 809 patients predominantly in Child–Pugh A and ECOG 0 stages, reported a median overall survival (OS) of 11.08 months and progression-free survival (PFS) of 3.24 months. The objective response rate (ORR) and disease control rate (DCR) were 10.1% and 65.5%, respectively. Regorafenib demonstrated an acceptable safety profile, with common adverse events such as diarrhea, fatigue, and hand–foot skin reaction. Severe adverse events occurred in less than 10% of patients. The findings support regorafenib as a valuable second-line therapy in advanced HCC and highlights regorafenib’s role in improving HCC management while identifying areas for future investigation.

In September 2020, following the results of the IMbrave150 trial [41], atezolizumab, an anti-programmed cell death ligand 1 (PD-L1) antibody, and in September 2020, the combination of atezolizumab, an PD-L1 antibody, and bevacizumab, a VEGF inhibitor, were approved and will become the first-line treatment for unresectable HCC by 2022. In addition, there is a new regimen following the results of the HIMALAYA trial [42], durvalumab + tremelimumab, a treatment with the anti-PD-L1 + anti-cytotoxic T-lymphocyte-associated protein 4 (CTLA-4) combination.

Atezolizumab + bevacizumab (atezo + bev) or durvalumab + tremelimumab (durva + treme) may be offered as first-line treatment for patients with Child–Pugh class A, and Eastern Cooperative Oncology Group performance status (ECOG PS) 0–1 advanced HCC by ASCO Guideline Update 2024 [43].

Blood flow imaging centered on dynamic CT has become indispensable not only for the qualitative diagnosis of tumors but also for determining treatment efficacy. In the future, it will be necessary to accumulate cases with issues in the evaluation of mRECIST and RECICL, and to discuss better criteria for determining efficacy, considering factors such as changes in tumor shape (internal necrosis), differences in efficacy between tumors, and the implications of dark staining. In addition, recently introduced immune checkpoint inhibitor (ICI) therapy for hepatocellular carcinoma, which has recently been introduced, causes unique changes in tumor size and blood flow after treatment, and may require different efficacy criteria than conventional local therapy or molecular-targeted drugs. Therefore, it is necessary to establish different criteria for determining the efficacy of ICI therapy compared with conventional local therapies and molecular-targeted drugs.

Kudo et al. [44] investigated whether objective response (OR), assessed via the modified RECIST (mRECIST) criteria, is a reliable surrogate endpoint and predictor of overall survival (OS) in patients with advanced hepatocellular carcinoma (HCC) undergoing systemic therapies [19]. The evaluation aims to bridge the gap in using radiological responses to guide treatment outcomes, considering the increasing complexity of sequential treatments in advanced HCC. A systematic review and meta-analysis were conducted on randomized clinical trials (RCTs) published between 2010 and 2020, encompassing 34 studies with 14,056 patients. These trials reported OR and OS using either RECIST or mRECIST criteria. Key findings reveal that the trial-level correlation between OR and OS was stronger for mRECIST (R = 0.677) compared to RECIST (R = 0.532). This indicates that mRECIST is more effective at capturing the radiological response reflective of survival outcomes. Moreover, patients achieving an OR as per mRECIST criteria demonstrated significantly improved OS, with a pooled hazard ratio of 0.44 (95% CI: 0.27–0.70). These results were particularly evident in patients treated with immune checkpoint inhibitors such as atezolizumab–bevacizumab, which outperformed tyrosine kinase inhibitors. Despite these promising findings, the study concludes that the correlation between OR and OS remains modest, limiting its application as a primary endpoint in Phase III trials. In conclusion, mRECIST demonstrates utility as an independent predictor of survival, providing critical insights into therapeutic efficacy in advanced HCC. However, its surrogacy for OS requires further validation through future trials with refined methodologies and more robust surrogate endpoints like progression-free survival.

**Figure 7:**
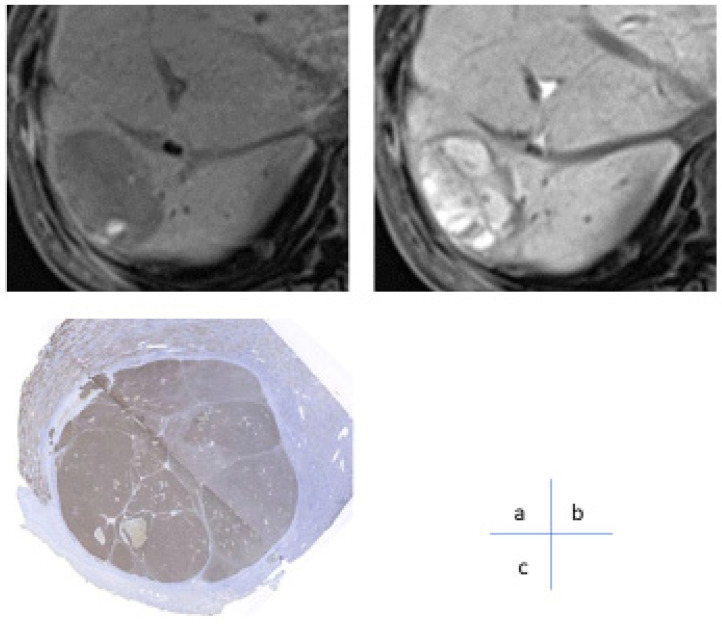
Hepatocellular carcinoma in the 60s showing increased EOB uptake in the hepatocellular phase. (**a**) A low-signal mass 4 cm in size can be noted on MRI T1-weighted image before contrast in S7 of the liver. (**b**) EOB uptake by the mass was enhanced over the surrounding liver parenchyma in the hepatocellular phase of Gd-EOB-DTPA contrast-enhanced MRI. (**c**) The surgical specimen shows intermediately differentiated hepatocellular carcinoma, and OATP1B3 immunostaining shows strong expression of OATP1B3 in the tumor.

Currently, there is no established biomarker for predicting the response to systemic chemotherapy in MTA exists because MTA has many targets beyond VEGF. Although perfusion MR/CT has been reported to be useful for predicting early response to treatment [45,46], it is not a common method for determining treatment response, and its clinical utility and impact have not been high. Gd-EOB-DTPA is a hepatocyte-specific contrast agent that is selectively taken up by hepatocytes via organic anion transporting polypeptide 1B3 (OATP1B3), a plasma membrane transporter. Catenin gene mutations and OATP1B3 have shown a strong correlation, and it has been reported that Wnt/β-catenin activating mutations can be detected with a sensitivity of 78.9% and specificity of 81.7% using the EOB-MRI hepatocyte phase [47]. Subsequent studies indicated that coactivation of Wnt/β-catenin mutations and their target gene, HNF4α, promotes the expression of OATP1B3 at the plasma membrane and depicts it as an iso to high signal nodule on EOB-MRI hepatocyte phase [48]. Among HCC with Wnt/β-catenin activating mutations, nodules with HNF4α expression can be recognized by EOB-MRI hepatocellular phase, and EOB-MRI can discriminate HNF4α-positive hepatocellular carcinomas with good prognosis with little vascular invasion or distant metastasis (Figure 7).

Furthermore, studies have indicated that HCCs with activated Wnt/β-catenin transmission pathways are resistant to first-line immune checkpoint inhibitor monotherapies. Our research group was the first to non-invasively identify HCC with Wnt/β-catenin activating mutations by EOB-MRI to predict therapeutic resistance to single ICI drugs [49]. Specifically, nodules showing high signal intensity in the hepatocellular phase of EOB-MRI (nodules with Wnt/β-catenin gene mutations) had a lower response rate to ICI monotherapy and significantly higher tumor growth and recurrence rates.

## 6. Conclusions

This review emphasizes advancements in imaging and systemic therapies for HCC. By integrating mRECIST, RECICL, and LI-RADS with new therapeutic strategies, this framework aims to enhance diagnostic accuracy and therapeutic outcomes in clinical practice.

## Figures and Tables

**Table 2:** Example table: comparison of mRECIST and RECICL.

Criterion	mRECIST	RECICL
**Measurement Method**	Unidimensional tumor measurement	Bidimensional measurement, including necrosis evaluation
**Application**	Locoregional and systemic therapies	Primarily locoregional therapies
**Evaluation Focus**	Viable tumor size reduction	Comprehensive assessment of necrosis and shrinkage

## Data Availability

The original contributions presented in this study are included in this article. Further inquiries can be directed to the corresponding author.

## Supplementary Materials

The following supporting information can be downloaded at: https://www.mdpi.com/article/10.3390/cancers17010101/s1, Figure S1a. Algorithm for surveillance and diagnosis (Algorithm 1); Figure S1b. Surveillance and diagnostic algorithm (Algorithm 2).
