# Supplementary material for: Radiological Assessment and Therapeutic Evaluation in Hepatocellular Carcinoma: Differentiation and Treatment Response with Japanese Guidelines
Source: Cancers (Basel). 2024 Dec 31;17(1):101. doi: 10.3390/cancers17010101 (PMC11719590; doi:10.3390/cancers17010101)
Supplement: Supplementary file 1 [file cancers-17-00101-s001.zip › cancers-3347352-supplementary.pdf]

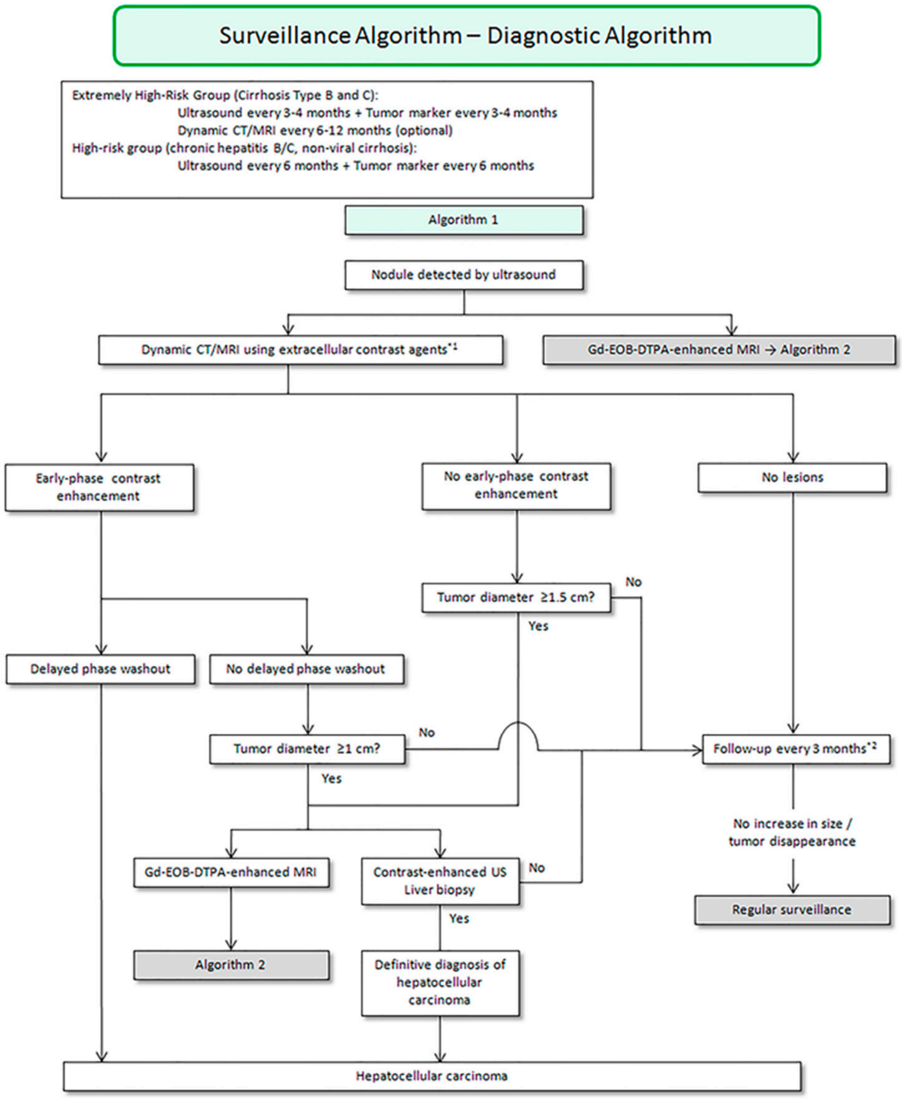

Figure S1a

a) Algorithm for surveillance and diagnosis (Algorithm 1)

Excerpt from Reference [1]

*1Dynamic computed tomography (CT)/magnetic resonance imaging (MRI) is used in some patients if the nodule(s) are not visualized on ultrasound (US) because of poor visualization and/or elevated levels of tumor markers (s). * 2 Lesions detectable on US were followed using US. Lesions undetectable on US may be followed-up with dynamic CT/MRI. Gd-EOB-DTPA: gadolinium-ethoxybenzyl-diethylenetriamine penta-acetic acid

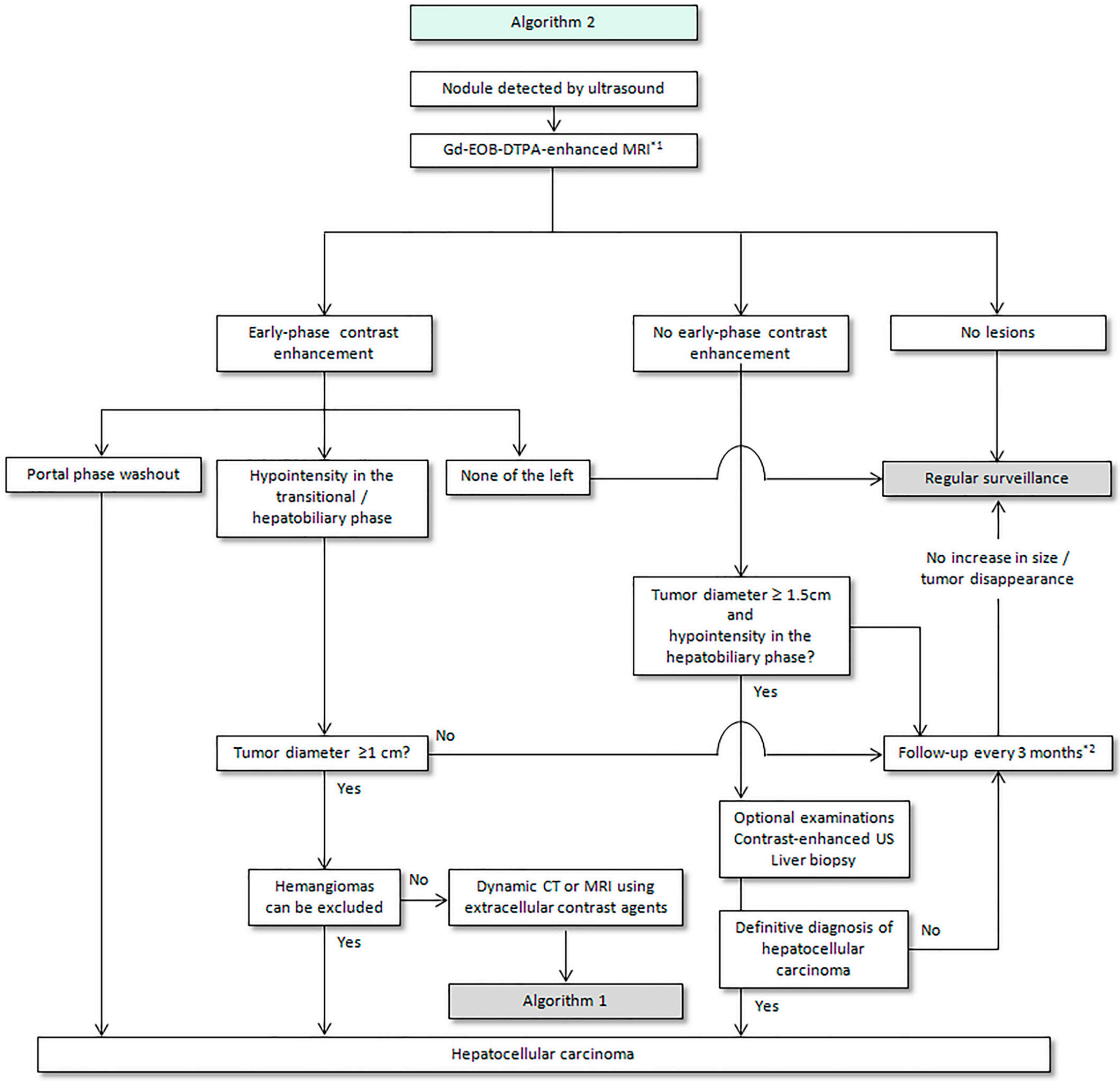

Figure S1b

b) Surveillance and diagnostic algorithm (Algorithm 2)

Excerpt from [1]

CT, computed tomography; Gd-EOB-DTPA, gadolinium-ethoxybenzyl-diethylenetriamine pentaacetic acid; MRI, magnetic resonance imaging
